# The Effects of *Gardenia Jasminoides* on Periodontitis in Ligature-Induced Rat Model

**DOI:** 10.3290/j.ohpd.a45084

**Published:** 2020-09-04

**Authors:** Dae Hoon Lee, Haesu Lee, Mi Hye Kim, Woong Mo Yang

**Affiliations:** a Researcher (PhD course student), Department of Convergence Korean Medical Science, College of Korean Medicine, Kyung Hee University, Seoul, Republic of Korea. Formal analysis; investigation; manuscript drafting, review, editing and revision.; b Researcher (PhD course student), Department of Convergence Korean Medical Science, College of Korean Medicine, Kyung Hee University, Seoul, Republic of Korea. Formal analysis; investigation; manuscript review, editing, and revision.; c Research Professor, Department of Convergence Korean Medical Science, College of Korean Medicine, Kyung Hee University, Seoul, Republic of Korea. Conceptualisation; investigation; methodology.; d Associate Professor, Department of Convergence Korean Medical Science, College of Korean Medicine, Kyung Hee University, Seoul, Republic of Korea. Conceptualisation; project administration and supervision.

**Keywords:** periodontitis, Gardenia jasminoides, alveolar bone loss, osteoclasts

## Abstract

**Purpose:**

Periodontitis is characterised by inflammation of periodontium and alveolar bone loss. *Gardenia jasminoides* is reported to have anti-inflammatory effects. In this study, we investigated the effects of aqueous extract of *G. jasminoides* (GJ) on periodontitis.

**Materials and Methods:**

Male Sprague-Dawley rats aged 7 weeks were randomly placed in three groups (n = 7); non-ligatured and non-treated (NL group), ligatured and distilled water-treated (L group) and ligatured and 100 mg/kg GJ-treated (GJ group). After oral administration of GJ for 14 days, the mandibles were removed for histology. In addition, RAW 264.7 cells were treated with 100 ng/ml receptor activator of nuclear factor-κΒ ligand (RANKL) and 1, 10 and 100 μg/ml GJ for 7 days to analyse the expression of periodontitis-related factors.

**Results:**

In GJ-treated mice, the score of alveolar bone loss was statistically significantly attenuated compared with the L group. GJ treatment showed inhibition effect in the progress of cementum demineralisation. The expressions of proinflammatory cytokines in gingival tissue were statistically significantly regulated by GJ treatment. Additionally, GJ treatment showed the dose-dependent inhibition of RANKL-induced osteoclast formation. Furthermore, GJ treatment downregulated the RANKL-induced cytokine production in RAW 264.7 cells.

**Conclusion:**

In summary, GJ ameliorated periodontitis-induced alveolar bone loss via inhibiting transcription factors including nuclear factor-κB, c-fos and extracellular signal-regulated kinase signalling. Therefore, GJ might be a therapeutic option for treating periodontitis.

Periodontitis is a chronic dental disease, characterised by inflammation of the periodontal structures that support and surround the teeth.^18^ A recent study demonstrated that a prevalence rate of periodontitis is 47.2% in adults of age 30 years and above in the US.^5^ Typical signs of periodontitis found in patients are cementum degradation, alveolar bone loss and destruction of gingiva.^6^ Since alveolar bone destruction is closely linked to teeth loss, inhibition of alveolar bone resorption could be a therapeutic target for periodontitis. However, current clinical treatment of periodontal diseases focuses antibacterial or anti-inflammatory effects such as metronidazole, doxycycline and ketoprofen.^1,3,10,13^ Therefore, alveolar bone remodelling-based approaches for periodontitis represent a novel alternative to conventional drugs.

*Gardenia jasminoides Ellis (Rubiaceae)* is used as a traditional medication in Korea, China and Japan.^19^ ‘Donguibogam’, a classic book of Korean medicine, describes that *G. jasminoides* (GJ) reduces fever, discharges inflammation, cools heat, eliminates vexation and serves as a diuretic. In addition, GJ was reported to act as an analgesic and possess anti-inflammatory, antiphlogistic, antipyretic and homeostatic effects currently.^9^ However, the efficacy of GJ on periodontitis is yet to be investigated. The aim of this study is to demonstrate the effects of GJ on periodontitis and determine its molecular mechanism.

## Materials and Methods

### Preparation of GJ

The fruit of *G. jasminoides* were obtained from Jung-do Herb (Seoul, Korea). *G. jasminoides* was extracted with distilled water (DW) for 1 h at 100°C. The extract was filtered, concentrated and lyophilised by freezing dryer. The yield of *G. jasminoides* was 13.48%. The final obtained powder of *G. jasminoides* water extract was called GJ. A voucher specimen was deposited at our laboratory.

### Experimental Design

Sprague-Dawley rats (7 weeks aged, male; RAONBIO, Yongin, Korea) were housed in an air-conditioned room (20 ± 2°C temperature and 50 ± 5% humidity) under a 12 h light/dark cycle with food and water freely available. All experiments were conducted according to the guidelines of the Guide for the Care and Use of Laboratory Animals of the National Institutes of Health. The protocol was approved by Committee on Care and Use of Laboratory Animals of the Kyung Hee University (KHUASP(SE)-16-045).

The rats were randomly placed in three groups (n = 7): (i) NL, non-ligatured and non-treated; (ii) L, ligatured and DW-treated; and (iii) GJ, ligatured and 100 mg/kg GJ-treated. Under general anaesthesia with intraperitoneal injection of a tiletamine/zolazepam mixture (Zoletil 50; Virbac Lab, Carroscedex, France), the experimental periodontitis was induced by a ligature (sterilised 3–0 nylon) placement into the subgingival sulcus around the both sides of mandibular first molar, except NL group. Immediately after ligature placement surgery, 100 μl of DW and GJ, dissolved in DW, were orally administrated for 2 weeks. Then, the rat was sacrificed to collect molars on both sides and gingival tissues.

### Measurement of Alveolar Bone Loss

The left mandibles were defleshed with boiled water and stained with 1% aqueous methylene blue (Sigma, MO, USA) for 5 min. Images were obtained from Smart Microscope Pro (Kangjin Technology, Seoul, Korea) with ×10 magnification. Three distances from the cementoenamel junction to the alveolar bone crest of the mesial, middle and distal sites on the buccal side were calculated by an Image J computerised densitometry system (NIH, Bethesda, MD, USA), respectively (n = 7). Three points were summed for each first molar as a score of alveolar bone loss, in mm.

### Histological Analysis

The right side of the mandibles were fixed in 10% neutral buffered formalin for 18 h and demineralised in a solution of 0.1 M ethylene diaminetetraacetic acid for 2 months. After dehydration with ethanol and xylene, the specimens were embedded in paraffin. Serial sections of 7 μm thickness were stained with haematoxylin and eosin (H&E). The integrity of periodontal structures was observed with ×40 and ×400 magnification. The digital images were obtained from Leica Application Suite microscope software (Leica Microsystems, Buffalo Grove, IL, USA). The lengths of gingival inflamed epithelium and cementum from cementoenamel junction of slides were chosen at random (n = 7) and measured by a computerised densitometry system, Image J (NIH, Bethesda, MD, USA).

### Measurement of Tartrate-Resistant Acid Phosphatase (TRAP) Activity

7 μm cut tissue sections were stained with tartrate-resistant acid phosphatase (TRAP; Sigma, MO, USA) and naphthol ASBI phosphate (Sigma, St Louis, MO, USA) to observe osteoclastic activity. To classify the TRAP-stained cells and nuclei, haematoxylin staining was performed. The TRAP-positive osteoclasts were revealed at ×400. In addition, RAW 264.7 cells were seeded in 12-well plates and allowed to adhere 6 h to differentiate into osteoclasts. Cells were grown at 37°C in an atmosphere containing 5% CO_2_ that 95% humidity. After removal of the medium, cells were treated with 100 ng/ml receptor activator of nuclear factor-κΒ ligand (RANKL) and 1, 10 and 100 μg/ml concentrations of GJ suspended in α-minimal essential medium with 10% heat-inactivated fetal bovine serum for 7 days. The medium was replaced every 3 days. Osteoclast differentiation was monitored using a TRAP staining kit. To quantify the TRAP activity, each well was added 400 μl of citrate solution. After 1 h, supernatant was collected and added 400 μl of 0.1 N sodium hydroxide and measured at 410 nm using a microplate reading instrument. The experiments were carried out three times in triplicate measurements.

### Enzyme-Linked Immunosorbent Assay (ELISA)

RANKL-induced osteoclast was prepared as just described. After 7 days to induce the differentiation of osteoclast, the supernatant was collected and centrifuged to obtain the pure medium. The concentrations of tumour necrosis factor (TNF)-α and interleukin (IL)-6 was measured by each commercial enzyme-linked immunosorbent assay kits (BD Biosciences, CA, USA). The experiment was carried out according to the manufacturer’s protocol. Cytokine levels were estimated at an absorbance of 450 nm using a microplate reading instrument.

### Western Blot Analysis

RANKL-induced osteoclast was prepared as just described. Radioimmunoprecipitation assay buffer (50 mM Tris–HCl (pH 7.4), 1% Nonidet P-40, 0.5% sodium deoxycholate, 150 mM NaCl) containing protease inhibitors (Roche, Hoffmann, USA) was used for cell protein extraction. After quantification using Bradford method, 20 μg of protein was denatured with sodium dodecylsulphate buffer, then electrotransferred onto a polyvinylidene fluoride membrane (Bio-Rad, Hercules, CA, USA). Following incubation with primary and secondary antibodies (Cell Signaling, USA), the proteins were detected using an enhanced chemiluminescence detection system (Amersham Pharmacia Biotech, Uppsala, Sweden). Relative band densities were determined using an Image J computerised densitometry system.

### Statistical Analysis

All data were expressed as mean ± standard error of mean (SEM). Statistical significance was determined by one-way analysis of variance and Dunnett’s multiple comparison tests. In all analyses, p <0.05 was taken to indicate statistical significance in all analyses.

## Results

*Gardenia jasminoides* inhibited the alveolar bone loss in ligature-induced periodontitis.

**Fig 1:**
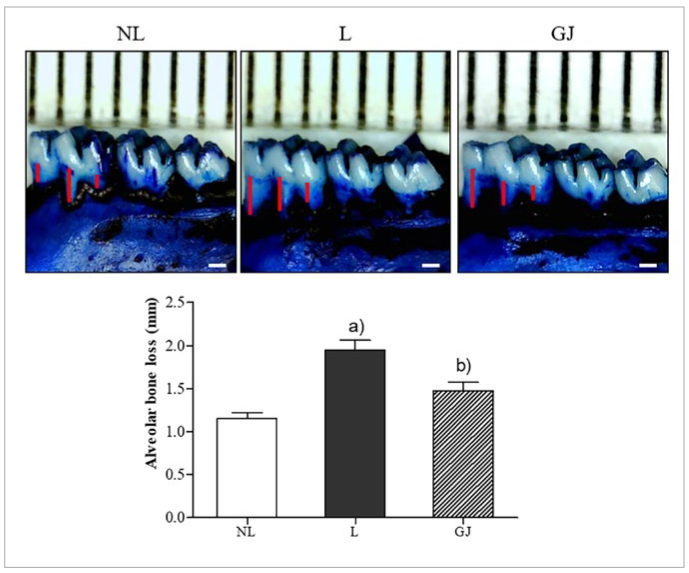
Effect of Gardenia jasminoides on alveolar bone loss indicated by methylene blue staining in ligature-induced periodontitis rat. Red lines represent the distance from the cementoenamel junction to the alveolar bone crest. NL, non-ligatured and non-treated; L, ligatured and DW-treated; GJ, ligatured and 100 mg/kg GJ-treated. Results are presented as mean ± SEM. (a) p < 0.05 in comparison with the NL group. (b) p < 0.05 in comparison with the L group.

In ligatured molar, the score of alveolar bone resorption was statistically significantly increased about 47.4% compared to non-ligatured normal group (NL = 1.09 ± 0.15 mm; L = 2.08 ± 0.29 mm, p <0.001). Administration of 100 mg/kg GJ reversed alveolar bone loss caused by periodontitis compared with L group (GJ = 1.40 ± 0.21 mm, p <0.01, Fig 1).

*Gardenia jasminoides* ameliorated the destruction of periodontium.

**Fig 2:**
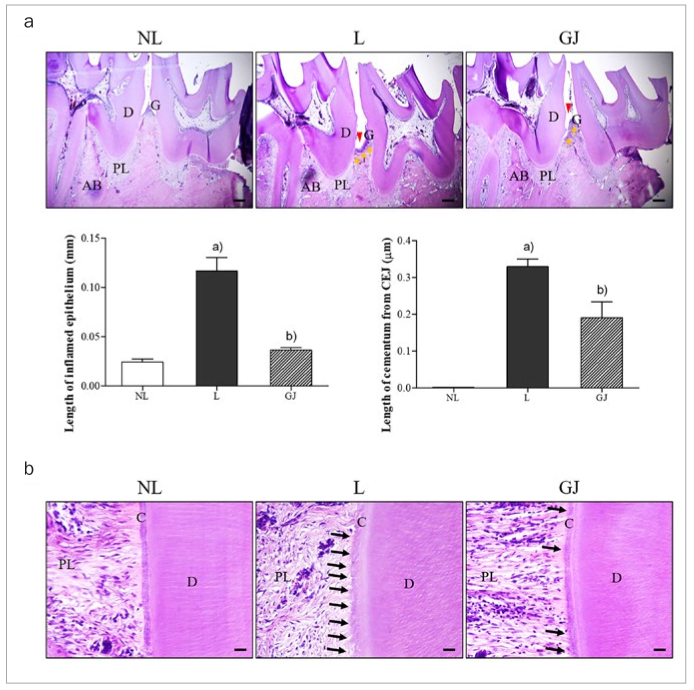
Effect of Gardenia jasminoides on histological changes of the periodontium indicated by H&E staining. Yellow arrows, inflamed epithelium in gingiva; red arrow heads, periodontal pocket; black arrows, root resorption pits lining the cementum surface. AB, alveolar bone; C, cementum; CEJ, cementoenamel junction; D, dentine; G, gingiva; PL, periodontal ligament. NL, non-ligatured and non-treated; L, ligatured and DW-treated; GJ, ligatured and 100 mg/kg GJ-treated. The scale bar is 1 and 0.1 mm in (a) and (b), respectively.

In ligature-induced periodontitis groups, increase of cementum from cementoenamel junction length, periodontal pocket, inflamed epithelium length and rough surface of cementum was exhibited compared to NL group (Fig 2a). Several roots’ resorption lining the cementum surface appeared in the impaired periodontium (Fig 2b). Administration of GJ ameliorated the symptoms of ligature-induced periodontal disease. Inflamed periodontal epithelium was effectively prevented by GJ, that GJ treatment decreased the thickness of epithelial layer expanded by gingival inflammation in periodontitis rat. In addition, the length of cementum from cementoenamel junction, next to the periodontal pocket, was statistically significantly diminished by GJ treatment. Furthermore, resorption of alveolar bone as well as destruction of gum were markedly recovered in the GJ-treated group. In addition, GJ treatment markedly inhibited the progress of cementum demineralisation compared with the ligatured group.

*Gardenia jasminoides* inhibited the osteoclast formation in the both of alveolar bone and RANKL-induced RAW 264.7 cells.

**Fig 3:**
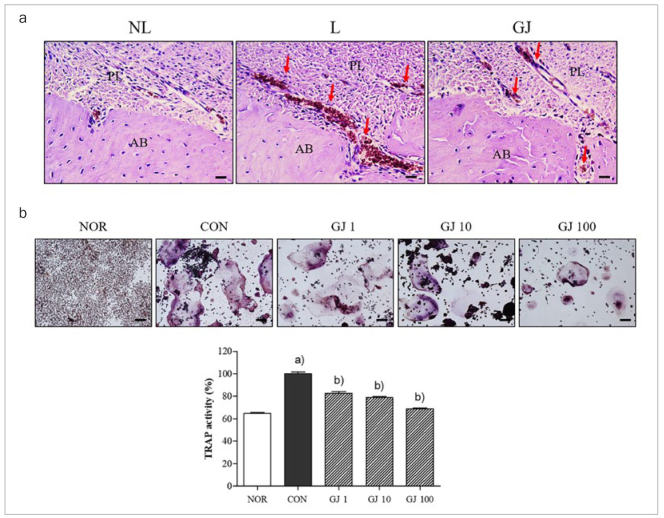
Effect of Gardenia jasminoides on osteoclast formation in ligature-induced periodontium and RANKL-induced RAW 264.7 cells. The red arrows indicate TRAP-positive osteoclast cells. AB, alveolar bone; PL, periodontal ligament. NL, non-ligatured and non-treated; L, ligatured and DW-treated; GJ, ligatured and 100 mg/kg GJ-treated. Results are presented as mean ± SEM. (a) p <0.05 in comparison with the NL group. (b) p <0.05 in comparison with the L group. NOR, non-treated cells; CON, RANKL alone-treated cells; GJ1, GJ10 and GJ100, RANKL with 1, 10 and 100 μg/ml GJ-treated cells. Results are presented as mean ± SEM. (a) p <0.05 in comparison with the NOR group. (b) p <0.05 in comparison with the CON group.

In the L group, numerous TRAP-positive cells were shown in the lesions of alveolar bone in periodontitis. The elevated TRAP-positive cells decreased by GJ treatment (Fig 3a). In addition, there was a statistically significant increase of mature multinucleated TRAP-positive osteoclasts in RANKL-stimulated RAW 264.7 cells. GJ treatment statistically significantly decreased osteoclast formation as shown in images by light microscope (Fig 3b). 1, 10 and 100 μg/ml GJ treatment showed the dose-dependent inhibition of RANKL-induced osteoclastogenesis (17.36%, 21.15% and 31.00%, respectively), as determined by measuring the cellular TRAP activity.

*Gardenia jasminoides* suppressed the RANKL-induced cytokine production in RAW 264.7 cells.

**Fig 4:**
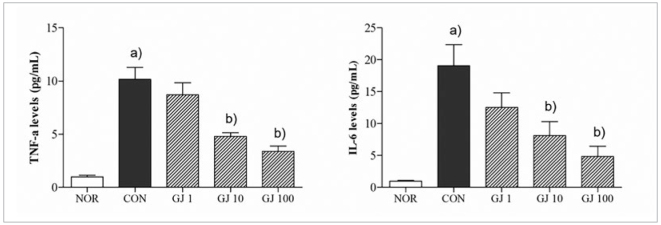
Effect of Gardenia jasminoides on RANKL-induced TNF-α and IL-6 production in Raw 264.7 cells. NOR, non-treated cells; CON, RANKL alone-treated cells; GJ1, GJ10 and GJ100, RANKL with 1, 10 and 100 μg/ml GJ-treated cells. Results are presented as mean ± SEM. (a) p <0.05 in comparison with the NOR group. (b) p <0.05 in comparison with the CON group.

RANKL stimulation in RAW 264.7 cells markedly induced the production of TNF-α and IL-6. 1, 10 and 100 μg/ml GJ treatment attenuated the increased levels of TNF-α and IL-6 in RANKL-induced osteoclast cells (Fig 4).

*Gardenia jasminoides* decreased the osteoclastogenesis-related factors in RAW 264.7 cells.

**Fig 5:**
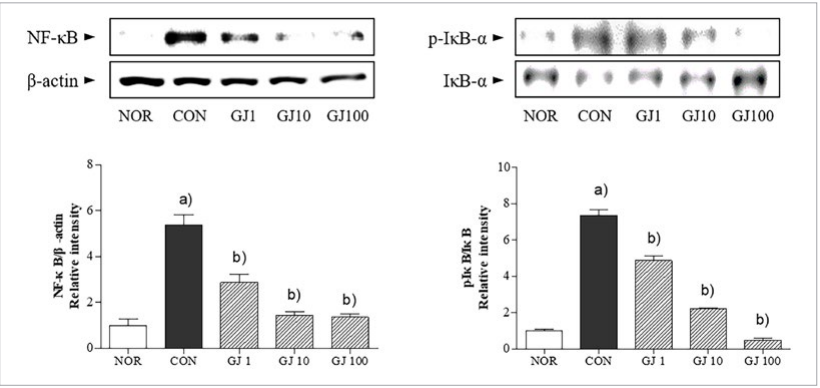
Effect of Gardenia jasminoides on RANKL-induced NF-κB activation in Raw 264.7 cells. NOR, non-treated cells; CON, RANKL alone-treated cells; GJ1, GJ10 and GJ100, RANKL with 1, 10 and 100 μg/ml GJ-treated cells. Results are presented as mean ± SEM. (a) p <0.05 in comparison with the NOR group. (b) p <0.05 in comparison with the CON group.

In osteoclasts induced by RANKL treatment, the protein expression of NF-κB was significantly 5.5 times increased. RANKL-induced NF-κB activation in RAW 264.7 cells was significantly reduced by GJ treatment at all concentrations. In addition, the phosphorylation of IκB-α was dose-dependently inhibited in the presence of GJ in osteoclasts, compared to only RANKL-treated cells (Fig 5).

**Fig 6:**
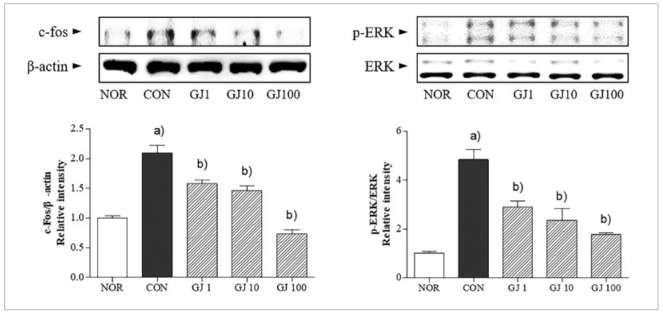
Effect of Gardenia jasminoides on RANKL-induced c-fos and ERK activation in Raw 264.7 cells. NOR, non-treated cells; CON, RANKL alone-treated cells; GJ1, GJ10 and GJ100, RANKL with 1, 10 and 100 μg/ml GJ-treated cells. Results are presented as mean ± SEM. (a) p <0.05 in comparison with the NOR group. (b) p <0.05 in comparison with the CON group.

The protein expression of c-fos was doubled in RANKL-induced osteoclast in comparison of non-treated cells. GJ reversed the increase of c-fos expression. Additionally, low, middle, and high concentration of GJ treatment dose-dependently inhibited the phosphorylation of ERK compared to only RANKL-treated control cells (Fig 6).

## Discussion

Alveolar bone loss is a crucial indicator of periodontitis development. The osteoclastic activity is excessively increased over the course of periodontitis, leading to alveolar bone loss.^4^ Therefore, inhibition of osteoclastic resorption in alveolar bone could be a valuable therapeutic approach to patients with periodontal diseases. In the present study, severe alveolar bone resorption was shown in rats with ligature-induced periodontitis. GJ treatment significantly alleviated alveolar bone loss in periodontal lesions. In addition, cementum, a bone-like connective tissue, undergoes excessive demineralisation with formation of osteoclastic resorption pits in periodontitis.^15^ GJ treatment recovered the demineralised structure of cementum in periodontal lesions. A previous report showed that chlorogenic acid derived from *G. jasminoides* inhibits lipopolysaccharide-induced periodontal bone loss via attenuating osteoclastogenesis.^12^ Based on this report, TRAP-positive osteoclasts were investigated in alveolar bone lesions to clarify the underlying mechanism on antiperiodontitis effects of GJ. We found that GJ administration decreased the TRAP-positive osteoclast formation in periodontal ligaments, demonstrating that GJ could attenuate the alveolar bone resorption by anti-osteoclastic effects.

To confirm the molecular mechanism on anti-osteoclastogenetic effects, inhibition of RANKL-stimulated osteoclastic activity by GJ was evaluated in RAW 264.^7^ cells. During alveolar bone resorption in periodontitis, osteoclastogenesis is promoted via multiple signalling processes such as activation of NF-κB and induction of c-fos followed by RANKL binding to receptor activator of nuclear factor-kappa B (RANK).^17^ GJ inhibited osteoclast formation not only in ligature-induced periodontitis, but also in RANKL-stimulated RAW 264.^7^ cells. In inflammatory osteoclastogenesis, TNF-α and IL-6 plays an important role on activation of macrophage colony-stimulating factor and RANKL.^11^ Increased TNF-α and IL-6 levels were suppressed in the presence of GJ in osteoclast-like cells.

NF-κB and inhibitor protein IκB-α are key factors in regulation of development, function and survival of osteoclasts.^8,16^ c-fos stimulates osteoclast differentiation by direct activation of nuclear factor of activated T cells 1.^2^ Therefore, regulation of osteoclastogenesis-related factors such as NF-κB and c-fos might be helpful to inhibit osteoclast formation. In addition, the binding of RANKL to RANK also simultaneously induces other signalling pathways such as mitogen-activated protein kinase pathways.^7^ The ERK pathway is associated with pivotal signalling for osteoclasts.^14^ In this study, GJ ameliorated NF-κB activation and phosphorylation of IκB-α. Also, c-fos expressions as well as ERK expression were apparently decreased by GJ treatment in RANKL-induced osteoclasts.

## Conclusion

Taken together, GJ prevented periodontitis-induced alveolar bone loss and demineralisation of cementum by inhibiting osteoclast formation. These anti-osteoclast effects of GJ are accompanied by the suppression of NF-κB, c-fos and ERK signalling pathways. Therefore, GJ might serve as a novel alternative for preventing periodontitis.
